# Mangrove response to environmental change in Australia's Gulf of Carpentaria

**DOI:** 10.1002/ece3.2140

**Published:** 2016-04-20

**Authors:** Emma Asbridge, Richard Lucas, Catherine Ticehurst, Peter Bunting

**Affiliations:** ^1^ Centre for Ecosystem Science School of Biological, Earth and Environmental Science The University of New South Wales High Street Kensington NSW 2052 Australia; ^2^ Commonwealth Scientific and Industrial Research Organisation (CSIRO) Christian Laboratory Land and Water Flagship Clunies Ross Street Black Mountain ACT 2601 Australia; ^3^ Department of Geography and Earth Sciences (DGES) Aberystwyth University Penglais Campus Aberystwyth Ceredigion SY23 3DB UK

**Keywords:** Biodiversity, botany, climate changes, ecology, ecosystems, mangroves

## Abstract

Across their range, mangroves are responding to coastal environmental change. However, separating the influence of human activities from natural events and processes (including that associated with climatic fluctuation) is often difficult. In the Gulf of Carpentaria, northern Australia (Leichhardt, Nicholson, Mornington Inlet, and Flinders River catchments), changes in mangroves are assumed to be the result of natural drivers as human impacts are minimal. By comparing classifications from time series of Landsat sensor data for the period 1987–2014, mangroves were observed to have extended seawards by up to 1.9 km (perpendicular to the coastline), with inland intrusion occurring along many of the rivers and rivulets in the tidal reaches. Seaward expansion was particularly evident near the mouth of the Leichhardt River, and was associated with peaks in river discharge with LiDAR data indicating distinct structural zones developing following each large rainfall and discharge event. However, along the Gulf coast, and particularly within the Mornington Inlet catchment, the expansion was more gradual and linked to inundation and regular sediment supply through freshwater input. Landward expansion along the Mornington Inlet catchment was attributed to the combined effects of sea level rise and prolonged periods of tidal and freshwater inundation on coastal lowlands. The study concluded that increased amounts of rainfall and associated flooding and sea level rise were responsible for recent seaward and landward extension of mangroves in this region.

## Introduction

Since 1980, at least 3.6 million ha of mangroves have been lost globally, primarily to agriculture and aquaculture, urbanization, and timber extraction (FAO, 2007). Gains in mangroves have also occurred, with these often following sediment delivery as a function of human‐induced land cover and use changes (Chimner et al. 2006; Lacerda et al. 2007; Stokes et al. 2010; Thomas et al. 2014; Godoy and Lacerda 2015). Natural events (e.g., cyclones, tsunamis) and processes (e.g., drought, flooding, sea level rise) have further changed the distribution and characteristic of mangroves. In many cases, these human‐induced and natural changes have occurred in combination, but their separation is often problematic (Field 1995).

Decoupling human‐induced and natural responses is desirable, particularly when evaluating the effects of climate change, and is best addressed by first considering the response of mangroves in areas relatively undisturbed from human activities (e.g., protected areas such as national parks or land that is far away from settlement and infrastructure; Thomas et al. 2014). One such area is the Gulf of Carpentaria in northern Australia, as mangroves along the coastline have remained relatively intact since European settlement (over 200 years ago). Hence, changes observed here can be attributed largely to a natural cause, acknowledging that some factors may still be exerting an indirect effect (e.g., changes in fire regimes and vegetation cover through grazing, erosion through cattle ranching). These mangroves may also indicate a response to impacts typically associated with climate change, namely changes in the amount and intensity of rainfall and sea level rise.

The aim of this research was therefore to quantify changes in mangroves along the coastline of the Gulf of Carpentaria and establish the main drivers of change. These included runoff as a function of rainfall variation, the frequency of freshwater inundation, and sea level fluctuation. The objectives were to: (1) map changes in mangroves by comparing time‐series classifications of Landsat sensor data acquired between 1987 and 2014; (2) focusing on the western Gulf (Leichhardt River), describe the impacts of change on forest structure using Light Detection and Ranging (LiDAR) and Advanced Land Observing Satellite (ALOS‐2) Phased Arrayed L‐band SAR (PALSAR‐2) acquired in 2012 and 2013; and (3) identify drivers of change by referencing existing rainfall, river discharge and sea level data and classifying inundation sequences using time series of Landsat and MODIS data.

## Study Area

**Figure 1:**
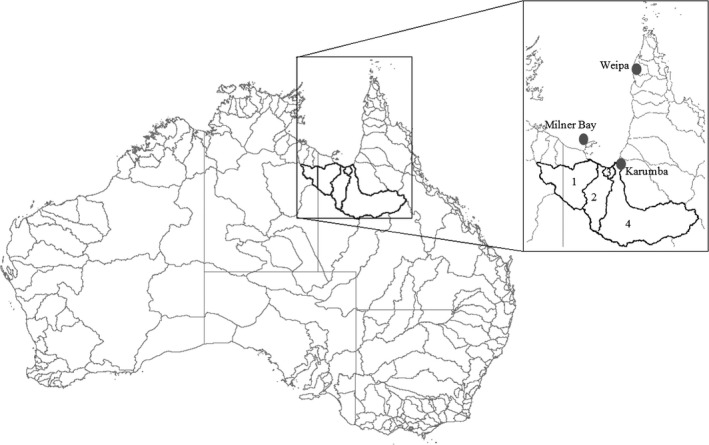
Location of the Nicholson (1), Leichhardt (2), Mornington Inlet (3), and Flinders River (4) catchments entering the Gulf of Carpentaria. Sea level monitoring stations are indicated by gray circles.

The Gulf of Carpentaria is located in northern Queensland and the Northern Territory, and mangroves are particularly prevalent along the coastlines of the Leichhardt, Nicholson, Flinders River, and Mornington Inlet catchments (Fig. 1). The predominant land use in the region is pastoral farming on freehold (19.5% of the land area) and leasehold (74.2%) land (Polidoro et al. 2010). The Gulf of Carpentaria has designated conservation areas and is protected within a marine national park zone and a special‐purpose zone.

The climate in the Gulf is hot and humid with “wet” and “dry” seasons. Rainfall increases toward the coast and varies from 800 mm/yr in the southern Gulf to >2000 mm/yr in the northern region. The rainfall is concentrated in the 4 months of the wet season (December through to March), producing runoff that is often beyond the river channel capacity. This exceptional rainfall leads to flooding which originates primarily from the Flinders River and expands into the lower reaches of the other catchments along the coast. The rivers collectively drain a ~0.5‐million‐km^2^ watershed which is relatively low‐lying (typically <20 m above mean sea level (msl)) (Prasad 2011). The wet season is also characterized by storm surges and cyclonic events, with an average of two to three cyclones occurring annually.

Mangroves are distributed along the majority of the Gulf coastline, typically as a narrow strip, with their establishment restricted to sheltered areas and shallow bathymetry (<80 m) as these prevent deep water waves and allow the forest to escape associated stressors (i.e., persistent erosion; Manson et al. 2005). The eastern Gulf includes arid and semiarid tidal flats and has a total mangrove area of 1609 km^2^ with an estimated 36 mangrove species including *Avicennia marina*,* Rhizophora stylosa*, and *Sonneratia alba* (Duke 2006). The expansive sediment accretion has also allowed numerous tidal wetlands to form around river mouths, which provide an ideal substrate for mangrove growth. Landward of the mangroves are wide (40 km) plains with chenier ridges which, in some regions, extend over 30 km due to fluvial deposition during flood events. Fluvial sediment supply (both suspended and bedload) into the Gulf is significant. Although the sediment supply is dominated by the Flinders River, which is one of the largest, it is also influenced by many of the small rivers that drain the large catchments. The diurnal mesotidal regime in the Gulf also exerts a strong control over the sediment dynamics, with an average range of 2–3 m. The exception is in the southeast where ranges often reach 4 m (Hopley and Smithers 2010).

## Methods

### Available data

To determine historical trends in rainfall, data were obtained from 1900 to 2014 from the Bureau of Meteorology (2014) online Climate Database. River discharge data were also obtained from all existing river gauge stations (Bureau of Meteorology, 2014), for the period 1968 to 2014 for the Flinders, Nicholson, and Leichhardt catchments to establish whether changes in rainfall corresponded to fluctuations in runoff. No data were available for Mornington Inlet. Daily sea level data were obtained from the National Tidal Unit (NTU) for three sites: Milner Bay (13° 51′ 36″ S, 136° 24′ 56″ E; 1980 to 2014), Karumba (17° 29′ 30″ S, 140° 50′ 0″ E; 1985 to 2012), and Weipa (12° 40′ 12″ S, 141° 51′ 48″ E; from 1985 to 2013). These data were used to evaluate the potential influence of sea level fluctuation to movement of mangroves along the coast and rivers and rivulets.

Landsat sensor data (85 cloud‐free scenes georeferenced to UTM Zone 54) were obtained from the United States Geological Survey (USGS) for the period 1984 to 2014. These data were processed subsequently to surface reflectance by the Queensland Department of Science, Information Technology and Innovation (DSITI). A bidirectional reflectance correction was also applied. Estimates of Foliage Projective Cover (FPC) were then generated using a multiple regression between field measurements of woody FPC and both Landsat sensor (including the Normalized Difference Vegetation Index (NDVI) and shortwave infrared channels) and climate data. FPC provides a measure of the vertical projection of vegetation within a pixel (Armston et al. 2009) and can therefore be used as a basis for classification. Given the relatively high cover, values typically exceed 80% for many mature mangroves. To capture the dynamics of flooding on a more frequent time step, near daily MODIS data were acquired at 500 m spatial resolution from 2000 to 2014.

LiDAR data (1 m post spacing; 24 strips approximating 1 km in width) were acquired over the mangroves at the mouth of the Leichhardt River in 2012 by Airborne Research Australia (ARA) and made available to the study. Orthorectified, slope‐corrected, and calibrated ALOS‐2 PALSAR‐2 ultrafine beam (UBD) L‐band HH and HV data (3 m resolution) were also provided for the Gulf coastline by the Japanese Space Exploration Agency (JAXA) through their Kyoto and Carbon (K&C) Initiative.

### Time‐series classifications of Landsat sensor data

Within each of the cloud‐free 85 Landsat scenes acquired during the flooding periods of 1987 to 2014, the extent of inundation was mapped by applying a supervised maximum‐likelihood classification (MLC) to the visible, near‐infrared and two shortwave infrared channels. This flood period encompassed the start to the end of each flood event, with the latter signified by complete drying of all inland water. This was necessary given that, after flood events, water often remained for several months on the area occupied by the low‐lying mudflats, saltmarshes, and mangroves. These classifications were then combined to generate a map of inundation frequency within the four catchments and over the period of the time series, recognizing that this was only partially complete as cloud‐free images alone were used.

The extent of mangroves was also extracted from a single classification from each year between 1987 and 2014. The time‐series classifications were used to determine the year when the canopy was sufficiently closed to allow detection from the Landsat sensor, while the frequency of occurrence over time indicated their age. An MLC algorithm (as described earlier) was applied, with indicative accuracies determined by comparing the 2000 classification with that generated for this year as part of the 2000 Queensland Mud Crab Survey (Hay et al. 2005). While mangroves are generally distinct from mudflats and salt flats within Landsat FPC data, these occurred in proximity to saltmarshes and other dune vegetation in the Leichhardt, Flinders, and Nicholson catchments; hence, their discrimination (e.g., based on thresholds of FPC) was difficult. However, for the Mornington Inlet catchment, mangroves were the only coastal vegetation and changes in FPC over time could therefore be compared. On this basis, a hexagonal grid layer (with each hexagon being 23 km in diameter) was overlain onto the FPC images and all lines crossing the coastline were selected to quantify the annual change in FPC values (and hence mangrove extent) in the landward and/or seaward directions. An FPC threshold of 10% was used to define the extent of mangroves, with this corresponding to a canopy cover of 20% (Asbridge and Lucas in review), and the frequency of occurrence of mangroves (based on pixels exceeding this threshold) was determined to indicate their landward and seaward progression. From the different transects, the rates of seaward and landward expansion per year were estimated.

### Structural classifications

From the LiDAR data acquired over mangroves at the mouth of the Leichhardt River, a Digital Terrain Model (DTM) and a Canopy Height Model (CHM) were generated at 1 m spatial resolution using SPDLib software and the procedures outlined by Bunting et al. (2013). To identify ground returns for use in the DTM generation, a Progressive Morphological Filter (PMF; Zhang et al. 2003) algorithm was applied in combination with the Multi‐Scale Curvature (MCC; Evans and Hudak 2007) algorithm. The CHM was then defined in relation to the DTM and interpolated using a natural neighbor algorithm. Canopy cover was also estimated from these data (Bunting et al. 2013).

The ALOS‐2 PALSAR‐2 UBD data were used in combination with the LiDAR CHM to indicate the extent of mangroves with and without prop root systems based on the method suggested by Lucas et al. (2007). In this approach, all mangroves with a height exceeding approximately 5 m (determined from LiDAR data) and with a low L‐band HH and HV return were linked with species with prop root systems, while all the remaining areas were dominated by other structural forms (e.g., those with pneumatophores).

### Open water maps from MODIS

To provide spatial maps of inundation extent at a higher (near daily) temporal frequency, MODIS Open Water Likelihood (OWL) data were generated for the four catchments. The OWL algorithm was developed by Guerschman et al. (2011) and uses the relationship between surface water (not obstructed by vegetation) and the MODIS Normalized Difference Vegetation Index (NDVI), the Normalized Difference Water Index (NDWI), and the shortwave infrared bands. Only OWL data corresponding to a small MODIS view angle were used (where the distance from pixel to sensor is less than 1000 km), with this based on recommendations by Ticehurst et al. (2014). Based on the number of pixels occupied by water, the total area and distribution of inundation within each catchment were determined to indicate patterns of water influx and retreat during and following flood events.

### Linking variables

For one or several catchments, information on the extent, structure, and/or species composition of mangroves was obtained from remote sensing data, while climate‐related variables (including sea level fluctuation) were obtained via direct measurement (e.g., rainfall and river gauge stations) or classification of Landsat or MODIS data (Table 1). Linkages were first made between climate and hydrological variables to establish the extent to which increases in rainfall led to corresponding changes in runoff. The extent to which hydrological changes and sea level fluctuation contributed to the observed changes in mangroves was then evaluated.

**Table 1:** An overview of the variables assessed in this study in order to quantify the changes in mangrove extent and species composition and establish potential natural drivers of change

Variable	Year	Description	Catchment				Source/reference
			L	F	N	M	
Vegetation extent, structure and/or species composition							
Mangrove extent and species composition	1987–2014	^c^Landsat imagery, 30 m resolution	●	●	●	●	DSITI, USGS
Mangrove extent	1987–2014	^c^Landsat Foliage Projective Cover (FPC)				●	DSITI
Digital Terrain Model and Canopy Height Model	2012	LiDAR, 2012, 1‐m grid cell	●				ARA
Mangrove species composition	2014	ALOS‐2 PALSAR‐2 L‐band, HH and HV, 3 m resolution	●				JAXA
Climate‐related and hydrological variables							
Rainfall	1984–2014	Daily	●	●	●		BOM
River discharge	1984–2014	Daily, monthly and annual	●	●	●		BOM
Open water and patterns of flooding and recession	2000–2014	MODIS: Open Water Likelihood (OWL) Algorithm, 500 m resolution	●	●	●	●	Guerschman et al. (2011) and Ticehurst et al. (2014)
Inundation frequency	1987–2014	Landsat imagery, 30 m resolution (85 images in total)	●	●	●	●	USGS
Sea level	1985–2014	Daily	Milner Bay, Karumba, Weipa				NTU

L = Leichhardt, F = Flinders, N = Nicholson, and M = Mornington inlet; ^c^supervised maximum‐likelihood classification. USGS, United Stated Geological Survey; DSITI, Department of Science, Information Technology and Innovation; JAXA, Japan Aerospace Exploration Agency; ARA, Airborne Research Australia; BOM, Bureau of Meteorology, Australia; NTU, National Tidal Unit.

## Results

### Changes in rainfall, runoff, and sea level

**Figure 2:**
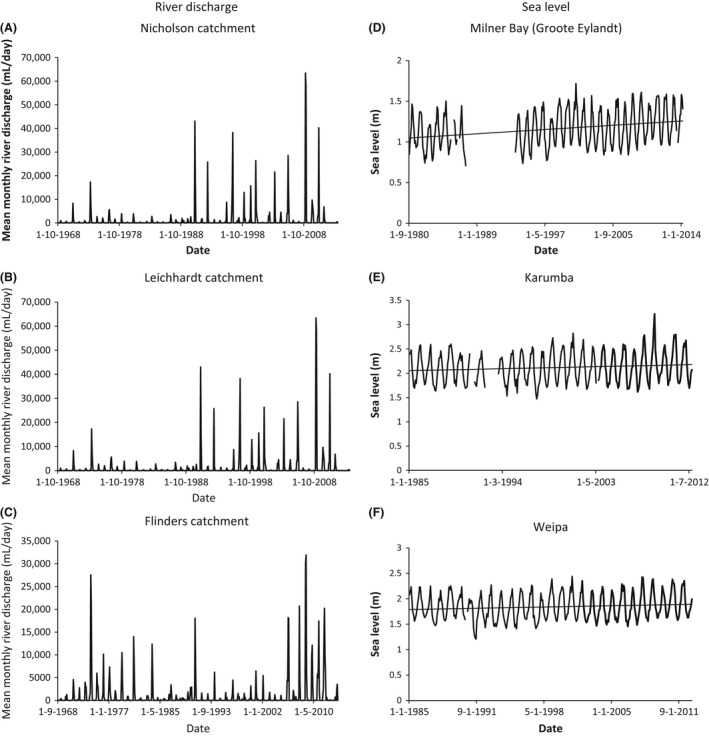
River discharge history for rivers within (A) the Nicholson, (B) Leichhardt and (C) Flinders catchments and sea level rise data for (D) Milner Bay, (E) Karumba, and (F) Weipa (Bureau of Meteorology, 2014, Department of Land Resource Management, 2014, Department of Natural Resources and Mines, 2014).

Over the period 1900 to 2010 (110 years), there were 12 major rainfall events in which the mean amount exceeded 400 mm/day, with four being in the last 20 years. River discharge (as measured at river gauges) was also generally low between 1900 and 1991, with only one major event (January 1974). However, the number and magnitude of peak discharges increased substantially thereafter for the Flinders, Nicholson, and Leichhardt catchments (Fig. 2A–C) in response to higher rainfall amounts. For example, the river discharge in the Leichhardt catchment was typically low (<250,000 mL/day) but nine peak discharge events above this threshold were recorded between 1968 and 2013, with each following large rainfall events. The largest of these were in January 1991, February 2009, and March 2011, with smaller events in January 1974 and December 2000. The timing of these peak river discharge events was similar across all three catchments (Fig. 2). The peaks in monthly river discharge in January 1991 and January/February 2009 for the Leichhardt catchment coincided with associated peaks in maximum rainfall recorded in this and the Flinders catchment and, in the latter case, the Nicholson catchment.

**Figure 3:**
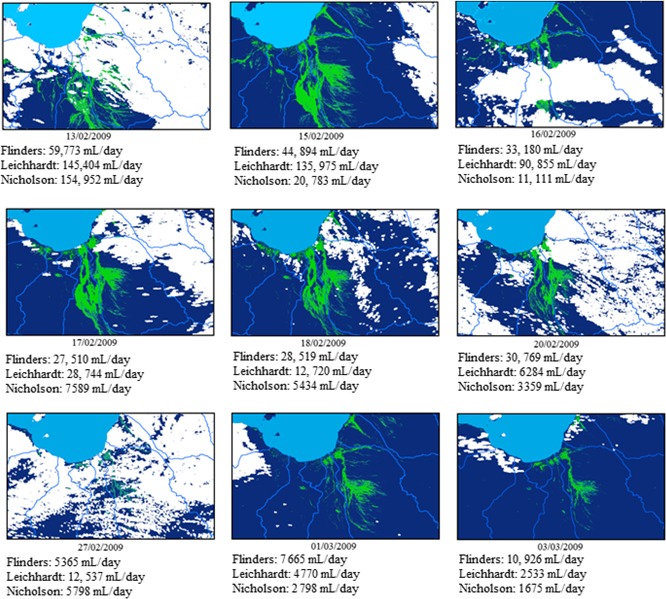
MODIS flow sequences of inundation and recession following the 2008/2009 major flood event. The river flow, which is predominantly in the Flinders catchment, is indicated in green, the land is dark blue with the catchment outlines in a lighter blue, and the white areas are cloud cover.

**Figure 4:**
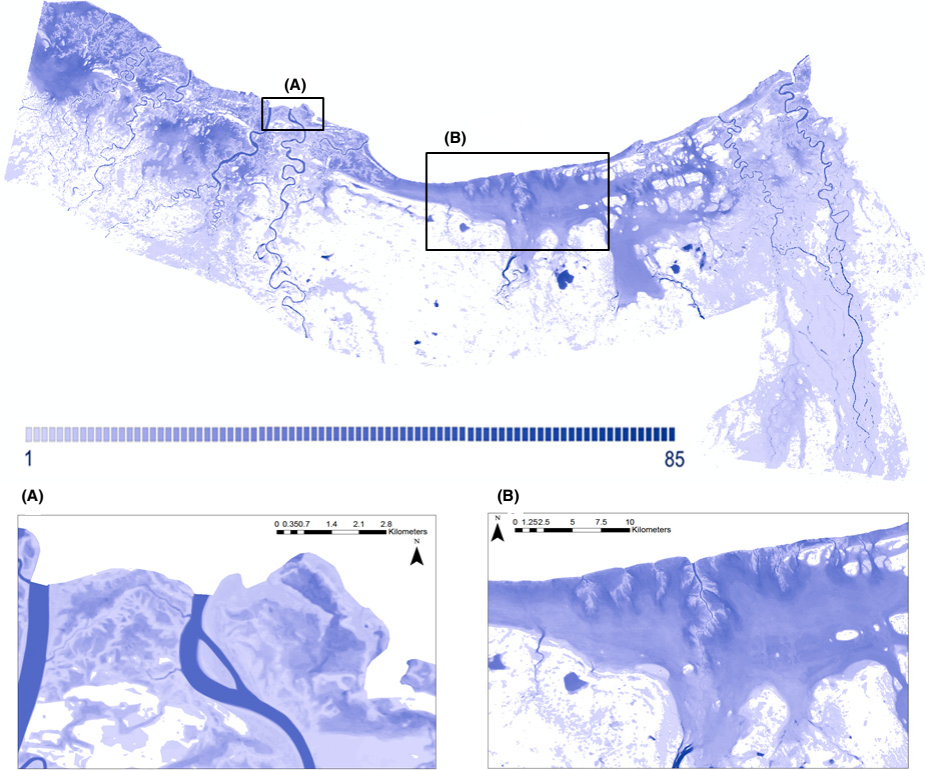
Open water map created using a supervised classification of 85 cloud‐free Landsat images from the start of the flood event to the end. Darker blues indicate more frequently inundated areas. Map inserts are (A) the mouth of the Leichhardt River and (B) the central coast (Mornington Inlet catchment).

The patterns of inundation and recession were also captured within the MODIS OWL data. Of particular note was the large fluvial inputs originating from the Flinders River and the rapidity by which the floodwaters discharged into the Gulf (Fig. 3; based on the 2008/2009 flood event). The persistence of accumulated water for several months in the low‐lying areas (below or close to 0 m above msl) of the near coastal zone, which were occupied primarily by mudflats and sand flats, was evident within both the MODIS and the time series of Landsat sensor data. The greatest frequency of inundation observed using the Landsat time series was in the Mornington Inlet catchment (inundated on 97.7% of cases) (Fig. 4), even though the majority of floodwaters originated from the large Flinders River toward the east. While direct rainfall inputs and freshwater runoff were the main contributors, these low‐lying areas were also flooded through tidal intrusion. The recession of floodwaters commenced from areas of high topography, with these often associated with those occupied by mangroves.

The NTU station data relating to sea level rise for Milner Bay (Groote Eylandt), Karumba, and Weipa for the period 1985 onwards indicate a steady increase (Fig. 2D and E), particularly at Milner Bay. Here, sea level increased by approximately 8.9 mm/year (National Tidal Unit, 2014).

### Delimiting mangrove forest age from Landsat

The overall accuracy in the classification of land cover and mangroves from the Landsat sensor data was 79% and 85%, respectively (based on a comparison of classifications from 2000 and the 2000 Queensland Mud Crab Survey data) with the latter being comparable to other studies (Ruiz‐Luna and Berlanga‐Robles 1999; Sulong et al. 2002; Giri et al. 2014). The mapping indicated that the majority of mangroves were present at the start of the time series (1987) and were hence older than 27 years. However, along the entire coastlines, mangroves consistently moved seaward or landward, including along the rivers and rivulets.

**Figure 5:**
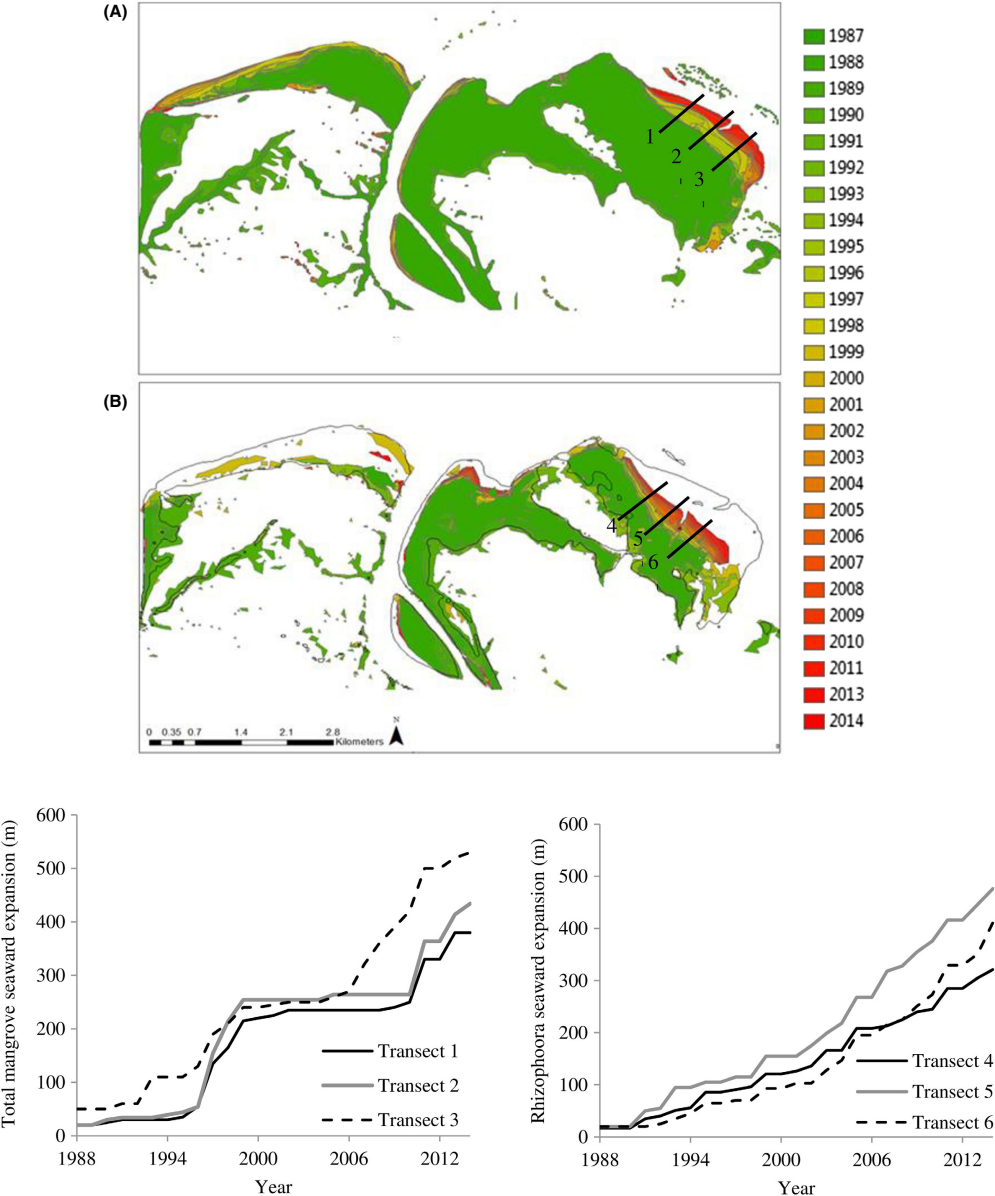
Changes in the extent of (A) all mangroves and (B) *R. stylosa* at the mouth of the Leichhardt River. The outline indicates total mangrove extent in 2014 and highlights the area of dieback on the landward margin. (C) The trend of yearly expansion in a seaward direction is shown for transects 1, 2, and 3 (all mangroves) and transects 4, 5, and 6 (mangroves dominated by *R. stylosa*).

The greatest rates of expansion were observed at the mouth of the Leichhardt River in 2011 and 2013, with the rate of movement being, on average, 195 m/year (±400 m) and 80 m/year (±20 m), respectively (Fig. 5A). The zone of mangroves dominated by *R. stylosa* at the mouth of the Leichhardt River also gradually extended in a seaward direction, from 1987 to 2014, into a zone dominated by *A. marina* (Fig. 5B). These rates of change are indicated in Figure 5C, based on transects extending across the area of seaward expansion and the zone dominated by *R. stylosa* which was steadily replacing mangroves dominated by *A. marina*.

**Figure 6:**
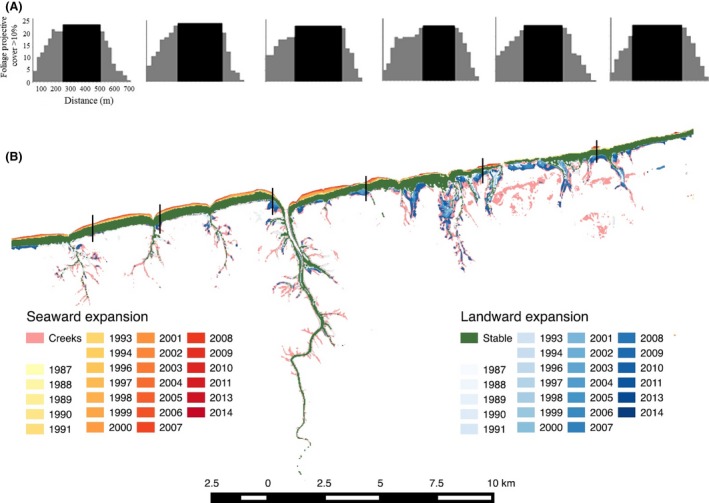
(A) The frequency of pixels along transects with a Foliage Projective Cover (FPC) >10%, highlighting both landward and seaward expansion of mangroves. (B) Map of mangrove change along the coastline of Mornington Inlet showing consistent expansion in a seaward direction and inland intrusion on the landward margins and along creeks.

Along the coastline of the Mornington Inlet catchment, the counts of pixels with a Landsat FPC exceeding 10% was used to indicate the seaward and landward expansion of mangroves between 1987 and 2014 (28 years, excluding 3 years because of Landsat‐7 sensor error). The central core zone of stable mangroves had an FPC count of 25 with lower values for the landward and seaward fringes experiencing expansion. In addition, most of the major creeks in this region were also experiencing significant landward expansion (Fig. 6).

### Quantifying change based on structure retrieved from LiDAR and SAR data

For mangroves occurring near the mouth of the Leichhardt River, five zones were discerned from age class maps generated using the Landsat sensor data, with these representing different stages of structural development (i.e., height and canopy cover). Zones 1 and 2 on the landward margins were associated with mangroves experiencing dieback between 1987 and 2014. The canopy cover derived from LiDAR was 82% for Zone 1 but >90% for the other zones, and the variation in cover was 4.1%. Despite the high canopy cover (99%), Zone 2 was classed as dieback because of the relatively high variation in cover (also 4.1%). Zone 3 was associated with mature forests older than 27 years and, based on the LiDAR data, the mean height approximated 10 m in height and the maximum height was 15.9 m. Zones 4 and 5 represented mangroves advancing on the seaward margins, with these being 5.4 m and 4.4 m in height, respectively. These regenerating zones were characterized by a low variation in height, with this being 1.6 and 1.3 m, respectively.

**Figure 7:**
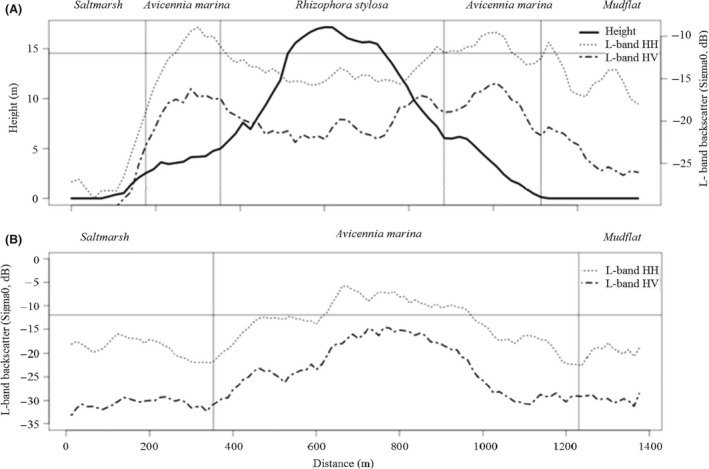
(A) Mangroves at the mouth of the Leichhardt River indicate a reduction in L‐band HH backscatter in areas of tall mangrove dominated by *R. stylosa* and *C. tagal*. The majority of mangroves dominated by *A. marina* exhibited an L‐band backscatter coefficient exceeding −12.0 dB. (B) Mangroves along the central Gulf coast (Mornington Inlet catchment), with the L‐band HH backscatter coefficient exceeding −12 dB in the central zone along a transect from the landward to the seaward margins.

Comparisons between the LiDAR and ALOS‐2 PALSAR‐2 data identified that the L‐band HH (horizontally transmitted and received) backscatter from older mangroves (i.e., those which established earlier) increased to about −9 dB with height up to about 4.5–5 m. However, beyond this height threshold, mangroves exhibited a lower backscatter (−9 to −16 dB). The L‐band HV backscatter also decreased from a maximum of −16 dB to a minimum of −22 dB. These differences are illustrated in Figure 7A, which represents data along a transect from the landward to the seaward margin at the mouth of the Leichhardt River. The low backscatter from high mangroves has been attributed to the presence of large prop root systems typical of species such as *R. stylosa* (Lucas et al. 2007) and is not observed where pneumatophores are prevalent (e.g., mangroves dominated by *A. marina*). On this basis, and with reference also to the 2000 Queensland Mud Crab Survey (Hay et al. 2005), the majority of mangroves responsible for the seaward migration at the mouth of the Leichhardt River are comprised of *A. marina* and other species of similar structure. Along the remainder of the Gulf coastline, a low backscatter at L‐band HH and HV was not evident (Fig. 7B), suggesting the predominance of species lacking prop roots. This was again confirmed through reference to the Queensland Mud Crab Survey mapping, which indicated that *A. marina* and species of similar structure were more widespread in this region and are associated with the landward and seaward movement observed.

### Linking climate and hydrological variables to mangrove change

#### River discharge and inundation versus seaward expansion

**Figure 8:**
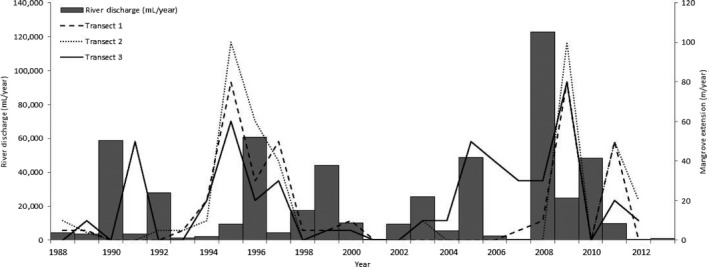
River discharge from 1988 to 2014 and the associated changes in mangrove area (plotted with a 2‐year lag) along transects, at the mouth of the Leichhardt River (the locations of the transects are shown in Figure 5).

Recent increases in the seaward expansion of mangroves at the mouth of the Leichhardt River were explained by surges in river discharge. In 1993 (2 years following the 1991 flood event), the mangroves at the Leichhardt River extended by an average of 63 m (maximum of 90 m) in a seaward direction. Similarly, following the 2011 and 2013 floods, mangroves continued to expand by an average of 72.3 m (maximum 175 m) and 42.9 m (maximum of 81.9 m), respectively. A sample cross‐correlation function (CCF) calculated within the R statistical package indicated a lag of 1 year between river discharge and an increase in mangrove width (r ranging from 0.45 to 0.56 for three transects extending from the seaward edge in 1987 and 2012; Fig. 8). In each expansion phase, the mangroves colonized *en mass*, with each cohort attaining a similar height and cover.

Along the remainder of the Gulf coastline, the CCF indicated a lag of 2 years between river discharge and an increase in mangrove area (for the Mornington Inlet, *r* = 0.486). However, the relationship between mangrove expansion in a seaward direction and river discharge was relatively weak, with this attributed to the dispersal of water throughout the lower floodplain rather than through a defined channel, as was the case for the Leichhardt River.

Comparisons of the maps of mangrove extent with those of water inundation indicated that the majority of mangroves were irregularly inundated with freshwater, although they would have experienced tidal flows (Fig. 4). This was attributed to their slightly raised elevation (the equivalent of a levee), achieved through accumulation of sediments in the dense root system and water rapidly receding from these higher areas.

#### Sea level rise and mangrove movement

**Figure 9:**
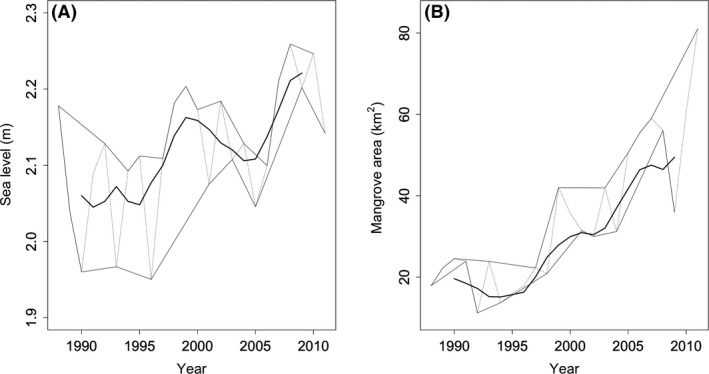
The trend in (A) sea level and (B) mangrove area over the time series (1987 to 2014).

A sample CCF was also used to identify the lag in mangrove area as a function of changes in sea level. The dominant cross‐correlations were evident with a lag of 1 year (0.52; varying by >0.4 for lags of 0 and 2 years). Based on a 1‐year lag (i.e., the rise in sea level in 1990 relates to the area of mangroves observed in 1991), the expansion of mangroves along seven of the eight creeks in Mornington Inlet broadly increased with sea level, as recorded at the closest gauge station of Karumba (Fig. 9). While sea level fluctuated widely, a general increase was observed when a centered moving average was considered. No erosion of mangroves from the coastal margin was observed, suggesting that rising sea levels are not leading to a loss of suitable habitat in this region.

## Discussion

### Seaward extension of mangroves

The seaward movement of mangroves was evident across the coastline of all four catchments and in particular at the mouth of the Leichhardt River and along the coastal margin of the Mornington Inlet. In the latter case, the expansion of mangroves occurred gradually throughout the assessment period, from 1987 to 2014, with no apparent links to river discharge. However, at the mouth of the Leichhardt River, significant seaward expansion of mangroves was observed approximately 2 years after the high discharge event, suggesting that this time is required for sediments to stabilize and propagules to establish. Following peak discharge in 1991, 2009, and 2010, mangroves colonizing the sediments at the mouth of the Leichhardt River were 3–4 m, 4–8 m, and 8–10 m in height in 2014 and the primary species was *A. marina*.

Maps of inundation extent from Landsat sensor and MODIS OWL data revealed a large amount of water spreading across the lower catchments of all rivers as well as the influence of tidal movement inland across a low‐lying coastal plain. The patterns of flood inundation and recession indicated that during a significant flood event, the rivers generally contributed proportionally to the area of inundation in the low‐lying coastal region (Mornington Inlet), with the largest being from the Flinders River. Much of the flooding was attributed to the monsoonal rains in the summer months. The change in the prevailing winds from a southeasterly to northwesterly direction from December to April also leads to an increase in tidal height, which is coincident with the stronger influence of the Madden–Julian Oscillation (Whitehouse 1943; Twidale 1956; Webber et al. 2010; Marshall and Hendon 2014). The associated inundation of the mudflats from both the sea and the land, particularly in the Mornington Inlet catchment, was considered responsible for the gradual inland expansion of mangroves.

During major flood events, as observed in 2000/2001 and 2008/2009, the Flinders River was observed to be a significant driving force delivering fresh water and fluvial sediment into the areas landward of the mangroves, with this occupied by saltmarshes and mudflats/sand flats. As such, the river was considered to be a primary driver of mangrove change in the coastal region. The Flinders River is the longest in Queensland (1004 km) and has a number of major tributaries such as the Cloncurry River and Corella River. During major flood events, the floodwaters originating from the Flinders River reach the coastal margin and spread out into the adjoining Mornington Inlet, Nicholson and, to a lesser extent, the Leichhardt catchment. This is evident in the Landsat and MODIS data, which show large discharges of water and sediments from the Flinders River and their distribution along the coast and across the catchments (Figs. 3 and 4). The floodwaters remain in the low‐lying coastal zone for a number of weeks following the event. Seaward expansion of mangroves may have been facilitated by the transport of sediments into the nearshore area during the flood and as the floodwaters receded, thus increasing the area available for propagule establishment (Eslami‐Andargoli et al. 2009; Stokes et al. 2010; Balke et al. 2015). Other studies such as Rakotomavo and Fromard (2010) in the Mangoky River delta, Madagascar, and Haworth (2010) in the Georges River, Australia, confirm that large river systems drive mangrove health and distribution in terms of sediment and propagule supply and inundation conditions. As the coastal lowlands are inundated for much of the wet season, the input of sediments is steady with some increases during major flooding events. Hence, the seaward expansion is more gradual and indirectly linked to discharge events.

Within the Gulf waters (on the seaward side), sediments are transported in accordance with tidal and wind‐driven currents. Tidal currents are predominantly clockwise traveling up to 3.0 km/h. Wind‐driven currents in the summer originate from the northwest, and in the winter from the southwest (Forbes and Church 1983; Woodroffe 1995). These currents lead to the redistribution (through erosion and deposition) of sediments (including those delivered through discharge events) in the nearshore area and increase the area available for mangrove propagules to colonize; hence, seaward expansion may be encouraged. The direction of the current is important as this controls the location of sediment deposition and therefore where propagules can establish (Lo et al. 2014).

### Landward expansion of mangroves

The inland intrusion of mangroves can be attributed, in part, to increased rainfall, which leads to persistent retention of freshwater on the landward margins. This creates conditions suitable for proliferation of mangrove forests (a lack of competition from terrestrial vegetation and a reduction in salinity) and thus enables them to better compete with saltmarsh for resources (Ebert 1995; Saintilan and Williams 1999; Rogers et al. 2005). While mangroves require saline environments, productivity and diversity often increase with the availability of freshwater input through precipitation, river flow, or runoff, because of the reduction in pore water salinity and associated saline stress (Pool et al. 1977; Cintrón et al. 1978; Ball and Pidsley 1995; Scavia et al. 2002; Parida et al. 2004). Mangroves are also likely to extend in a landward direction toward regions of reduced physiological stress (e.g., reduced inundation and soil salinity) (Rogers et al. 2006; Eslami‐Andargoli et al. 2009; Doyle et al. 2010; Krauss et al. 2011; López‐Medellín et al. 2011; Smith et al. 2013). Even so, optimum saline concentrations vary among species (Krauss and Allen 2003; Ye et al. 2005; Zaman et al. 2013). This may explain why the Mornington Inlet catchment has a greater incidence of progressive landward movement of mangroves due to more persistent inundation during the wet season, when compared to the mouth of the Leichhardt River where the floodplain drains at a faster rate.

The landward expansion of mangroves in the Mornington Inlet was not significantly correlated to river discharge. This may be due to the low‐lying nature of the region which is frequently and persistently inundated as a consequence of tides, rising sea levels and rainfall. Therefore, a peak river discharge event has a lesser impact as the land is consistently inundated. Even so, continued inundation during the wet season in particular encourages the gradual inland extension of mangroves.

Inland intrusion correlated with sea level rise, with this being as high as 8.9 mm/year in the Gulf Region (Gilman et al. 2008). In response to increased sea level, studies in Kakadu National Park (KNP) using time–time‐series comparison of aerial photography, hyperspectral airborne and LiDAR data, and also Landsat sensor data indicated that mangroves advance during periods of rising sea levels but maintain a foothold when these levels become lower. Hence, when a subsequent phase of sea level rise occurs, these mangroves are sufficiently well established to continue their colonization (albeit after some declines) in the landward direction, which is then progressive (Asbridge and Lucas in review). This same process is considered to occur in the Mornington Inset, which explains the fluctuation in mangrove area but overall increase in the seaward direction.

In other regions of the World, inland intrusion of mangroves has often been coupled with removal at the coastal margin, with this attributed to the slow rate of sediment accumulation relative to rises in sea level (Semeniuk 1994). As an example, the rate of sea level rise in Bermuda (28 ± 18 cm per 100 years; Pirazzoli 1986) is significantly greater than the rates of sediment accumulation. This has resulted in the erosion and retreat of mangroves on the seaward fringe (Ellison 1993). Persistent seawater flooding has also been found to reduce the capacity of leaf gas exchange (Krauss et al. 2006), leaf water potential and stomatal conductance (Naidoo 1983).

### Why do changes in the species composition occur?

The changes in the mangrove species composition in the Leichhardt River catchment suggested more favorable conditions for *R. stylosa* on the seaward margin of its zone; *A. marina* also progressively colonized the newly formed mudflats. However, conditions also became less suitable for *R. stylosa* on the landward margins, as evidenced by the greater canopy openness observed from LiDAR data and associated dieback. A similar process was also observed by Lucas et al. (2002) for Kakadu National Park where there was a distinct height difference between the 22 m tall *R. stylosa* forests and <5 m tall *A. marina* forests on the landward margins. In this case, conditions became unfavorable on the landward margin as the mangrove community as a whole progressively extended seaward leading to significant dieback of *R. stylosa*.

The observed change in species composition may be driven by the varying tolerances to the period and frequency of inundation, salinity, and sediment dynamics (Naidoo et al. 2002; Ye et al. 2005; Das 2013). For instance, *A. marina* has a wide tolerance to inundation and salinity, and this physiological advantage enables seaward expansion at the mouth of the Leichhardt River and along the Mornington Inlet as these areas are influenced by both the tidal regime and periodic freshwater river discharges. With forests dominated by *A. marina* extending in a seaward direction at the mouth of the Leichhardt River, those dominated by *R. stylosa* on the landward side are able to extend into the zone of *A. marina* and replace this species. These observations suggest that changes in the extent of different species types, growth stages, and structures also occur in addition to changes in extent suggesting the need to quantitatively describe the major biophysical elements of mangroves when developing monitoring systems.

### Compounding factors: increased frequency and intensity of storm events

The most widely accepted future trajectory for tropical cyclones in Australia is an increase in intensity and a shift southwards (Leslie et al. 2007; Abbs 2012; IPCC, 2013), although there is considerable variation. For instance, Walsh et al. (2004) predict a 56% increase in cyclone intensity by 2050, whereas (Abbs 2012) predict a 60% and 140% increase for 2030 and 2070, respectively. If the predictions are accurate with regards to intensity, this will lead to significant mangrove destruction as a result of increased wind speeds, inundation, sedimentation, and salinization but also opportunities for colonization or replacement of species. For example, stands dominated by *R. stylosa* are particularly vulnerable to storm damage and are slow to recover (Stoddart 1971; Woodroffe and Grime 1999). In this way, the mangrove community in the Gulf may change with an increasing occurrence of *A. marina* and a retreat of *R. stylosa*. Such changes in species may therefore counteract those associated with changing rainfall, discharges, inundation frequencies, and sea level fluctuation.

### Future response of mangroves to climate change

During the Holocene, mangroves were able to adapt to substantial change in climatic and hydrological conditions by expanding across available land; thereby indicating significant resilience (Woodroffe 1992; Alongi 2015). However, the current and future rates of climate change are unprecedented (Alongi 2014), with the outcomes including an increase in the frequency and also intensity of major rainfall events and a rise in sea level associated with thermal expansion of oceans and contributions to volumes through melting icecaps and glaciers. On the basis of current observations, both seaward and landward migration of mangroves is likely to continue. However, changes in the species composition that occur because of different tolerances to salinity and sediment loads will dictate the progress of expansion in both directions. Depending on local climatic and environmental conditions, and species composition, some mangroves forests may be able to survive the predicted changes in climate, as the trees are adapted to persist in a comparatively harsh environment. For instance, *A. marina* shows some level of tolerance to persistent inundation, and this may indicate the resilience of *A. marina* to sea level rise. However, there will be an upper tolerance limit and this is likely to vary with other variables such as topography, rainfall, sediment dynamics, and tidal regime. Further research is needed into species‐specific responses in order to predict the future diversity of mangrove forests.

## Conclusions

The study found that phenomena often associated with a changing climate (increased frequency and intensity of rainfall and associated flooding as well as sea level rise) are impacting on the extent, structure, and species composition of mangroves within the Gulf of Carpentaria, northern Australia. The primary conclusions are as follows:

1. Substantive seaward expansion of mangroves is occurring at the mouth of the Leichhardt River and along the coastline of Mornington Inlet. In the former case, mangroves benefit from the discharge of sediments to the nearshore environment through peak flooding events, which have increased in frequency (in 1991, 2009, and 2011) and intensity. Colonization of sediments occurred approximately 2 years after each flooding event. Seaward expansion along the Mornington Inlet was gradual and was linked to prolonged inundation of the low‐lying coastal zone from tidal flows and floodwaters, primarily from the Flinders River. These combined to continually pulse sediments into the nearshore, providing favorable conditions for mangrove expansion.
2. Movement of mangroves inland from the coast and along creeks was concentrated within Mornington Inlet and was attributed to the accumulation of water in the floodplain and rises in sea level. Persistent freshwater inundation on the landward margins during the wet season made conditions more suitable for mangroves by reducing salinity and allowing the forests to compete with saltmarshes and expand landwards.
3. Movement of species (primarily *R. stylosa*,* C. tagal*, and *A. marina*) within the mangrove zone was evident reflecting their different adaption to more or less favorable conditions. This observation reinforced the need for ongoing monitoring of the species composition and structure of mangroves as well as extent.
4. Other factors may also influence the changing distribution of mangroves, including air temperature, ocean circulation, microtopography, past storm events, wind magnitudes and patterns, and species‐specific physiochemical responses. To better understand the dynamics of mangroves in this region, a modeling approach that considers these drivers of change is needed.
5. As the area is relatively undisturbed from human activities, these mangroves are responding to natural events and processes, including those associated with a changing climate induced by human activities. Further monitoring of the dynamics of these mangroves is therefore recommended as they have been shown to be useful barometers of environmental change and can be used to gauge responses in an area far away from human activities.

## Conflict of Interest

None declared.
